# Indoleamine 2,3-dioxygenase 1 and overall survival of patients diagnosed with esophageal cancer

**DOI:** 10.18632/oncotarget.25235

**Published:** 2018-05-04

**Authors:** Ari J. Rosenberg, Derek A. Wainwright, Alfred Rademaker, Carlos Galvez, Matthew Genet, Lijie Zhai, Kristen L. Lauing, Mary F. Mulcahy, John P. Hayes, David D. Odell, Craig Horbinski, Srinadh Komanduri, Marie-Pier Tetreault, Kwang-Youn A. Kim, Victoria M. Villaflor

**Affiliations:** ^1^ Robert H. Lurie Comprehensive Cancer Center, Northwestern University, Chicago, 60611 IL, USA; ^2^ Department of Neurological Surgery, Feinberg School of Medicine of Northwestern University, Chicago, 60611 IL, USA; ^3^ Department of Preventive Medicine, Feinberg School of Medicine of Northwestern University, Chicago, 60611 IL, USA; ^4^ Department of Medicine, Feinberg School of Medicine of Northwestern University, Chicago, 60611 IL, USA; ^5^ Department of Radiation Oncology, Northwestern University, Chicago, 60611 IL, USA; ^6^ Department of Thoracic Surgery, Northwestern University, Chicago, 60611 IL, USA; ^7^ Department of Pathology, Northwestern University, Chicago, 60611 IL, USA; ^8^ Department of Gastroenterology, Northwestern University, Chicago, 60611 IL, USA; ^9^ Division of Hematology and Oncology, Northwestern University, Chicago, 60611 IL, USA; ^10^ Northwestern Medicine Developmental Therapeutics Institute, Chicago, 60611 IL, USA

**Keywords:** esophageal cancer, indoleamine 2,3 dioxygenase, immunotherapy, checkpoint inhibitor, the cancer genome atlas

## Abstract

**Background:**

Indoleamine 2,3-dioxygenase 1 (IDO1) is an enzyme with immunomodulatory properties that has emerged as a potential immunotherapeutic target in human cancer. However, the role, expression pattern, and relevance of IDO1 in esophageal cancer (EC) are poorly understood. Here, we utilize gene expression analysis of the cancer genome atlas (TCGA) and immunohistochemistry (IHC) to better understand the role and prognostic significance of IDO1 in EC.

**Results:**

High IDO1 mRNA levels were associated with worse overall survival (OS) in both esophageal squamous cell carcinoma (SCC) (*P* = 0.02) and adenocarcinoma (AC) (*P* = 0.036). High co-expression of IDO1 and programmed death ligand 1 (PD-L1) was associated with worse OS in SCC (*P* = 0.0031) and AC (*P* = 0.0186). IHC for IDO1 in SCC showed a significant correlation with PD-L1 (*P* < 0.0001) and CD3ε (*P* < 0.0001).

**Conclusions:**

EC with high IDO1 and PD-L1 expression is significantly correlated with decreased patient survival, and may correlate with increased T-cells. These data suggest that simultaneous inhibition of IDO1 and PD-(L)1 may overcome important barriers to T-cell mediated immune rejection of EC.

**Materials and Methods:**

mRNA expression data from TCGA (SCC *N* = 87; AC *N* = 97). IHC in a second cohort of EC (*N* = 93) were stained for IDO1, PD-L1, and CD3ε, followed by light microscopic analysis.

## INTRODUCTION

Esophageal cancer is a major cause of morbidity and mortality in the United States and worldwide. In 2017, an estimated 16,940 new cases and 15,690 deaths are expected due to esophageal cancer in the United States [1]. While the incidence of squamous cell carcinoma (SCC) of the esophagus is declining, esophageal adenocarcinoma (AC) and tumors of the gastroesophageal junction (GEJ) are increasingly common [1]. Despite advances in the treatment of esophageal and GEJ cancer, overall 5-year survival remains dismal [2]. Treatment for locally advanced disease typically includes a combination of chemotherapy and radiation, followed by surgical resection. Treatment for metastatic disease consists of palliative chemotherapy alone [2]. Targeted therapies have been extensively explored in esophageal and GEJ cancers, but with very limited success [3–8]. Trastuzumab, a monoclonal antibody that targets epidermal growth factor receptor 2 (Her2), is an option for the minority of patients with esophageal AC and GEJ tumors that overexpress Her2 [6]. Ramicurumab is a monoclonal antibody that targets vascular endothelial growth factor receptor (VEGFR) and offers a marginal benefit [7, 8]. Despite these small steps towards improved systemic therapy options in advanced esophageal and GEJ cancer, there is critical clinical need to improve therapeutic efficacy.

The recent success of immune checkpoint blockade in enhancing survival for a variety of cancers including melanoma, non-small cell lung and renal cell has sparked interest in its potential application for esophageal tumors. Immunotherapies that boost T cell efficacy aimed at the destruction of cancer cells have generated excitement for utilizing the release of endogenous immune response to control malignant progression. In particular, the inhibition of PD-1 and cytotoxic T lymphocyte antigen-4 (CTLA-4), has demonstrated clinical benefit in a number of malignancies [9] such as melanoma [10, 11], lung cancer [12], bladder cancer [13], kidney cancer [14], head and neck cancer [15], hepatocellular carcinoma [16], merkel cell carcinoma [17], Hodgkin lymphoma [18], gastric cancer [19], and microsatellite instability – high (MSI-high) tumors [20]. Combination checkpoint inhibition of PD-1 with CTLA-4 has been evaluated in melanoma, leading to a higher rate of objective response, progression free survival (PFS) and marginally overall survival (OS) than with anti-PD-1 alone, albeit with greater levels of toxicity [21, 22]. In advanced gastroesophageal cancer, response rates to single-agent checkpoint inhibitors in patient populations unselected for PD-L1 range from 11%–17%, while the response rate in patients selected for PD-L1^+^ tumors range from 13%–30% [19, 23–26]. In combination with CTLA-4 inhibition, response rates as high as 26% in unselected and 44% for PD-L1^+^ tumors have been observed [23]. Although these results with checkpoint inhibitors are exciting and encouraging leading to FDA approval, strategies to improve overall response rates, duration of response, and OS are needed.

A number of immunosuppressive factors associated with immune tolerance within the tumor microenvironment are being investigated as potential therapeutic targets to further amplify the antitumor activity mediated by checkpoint inhibition. One of these factors, indoleamine 2,3-dioxygenase 1 (IDO1), is overexpressed in a number of human malignancies in both tumor and stromal tissue, and is a major contributor to cancer-induced immune evasion [27]. IDO1 mediates the catalysis rate-limiting step in tryptophan degradation converting tryptophan into kynurenine [28], and demonstrates negative immunomodulatory properties [29]. IDO1 suppresses T-cell activity mediated by downstream stress response such as general control non-depressible 2 (GCN2) pathway and mTOR [29]. Depletion of tryptophan by IDO1 induces T-cell apoptosis and dysfunction [30], while accumulation of kynurenines are thought to induce immunosuppressive regulatory T-cells (Tregs). Further work has demonstrated that IDO1 expression is associated with inflammation within tumors [31]. IDO1 is induced by the pro-inflammatory cytokine, IFNγ, which also enhances expression of other key regulatory molecules including PD-L1 [32]. Patients treated with checkpoint inhibitors, such as PD-1 blockade, tend to have more robust clinical response in the setting of increased pre-treatment tumor-infiltrating lymphocytes [33]. Collectively, these findings support the hypothesis that the inhibition of IDO1 may increase intratumoral inflammation and increase tumor susceptibility to checkpoint inhibition, making it an ideal target for combining with the inhibition of PD-1 and/or CTLA-4. To the best of our knowledge, IDO1 expression in esophageal and GEJ tumors has not been extensively investigated. In this manuscript, we investigate the role, expression pattern, and relevance of IDO1 in esophageal cancer via gene expression analysis of the cancer genome atlas (TCGA) and quantitative immunohistochemistry (IHC) of surgically resected esophageal tumors.

## RESULTS

### IDO1 expression is associated with worse patient survival in esophageal cancer

**Figure 1:**
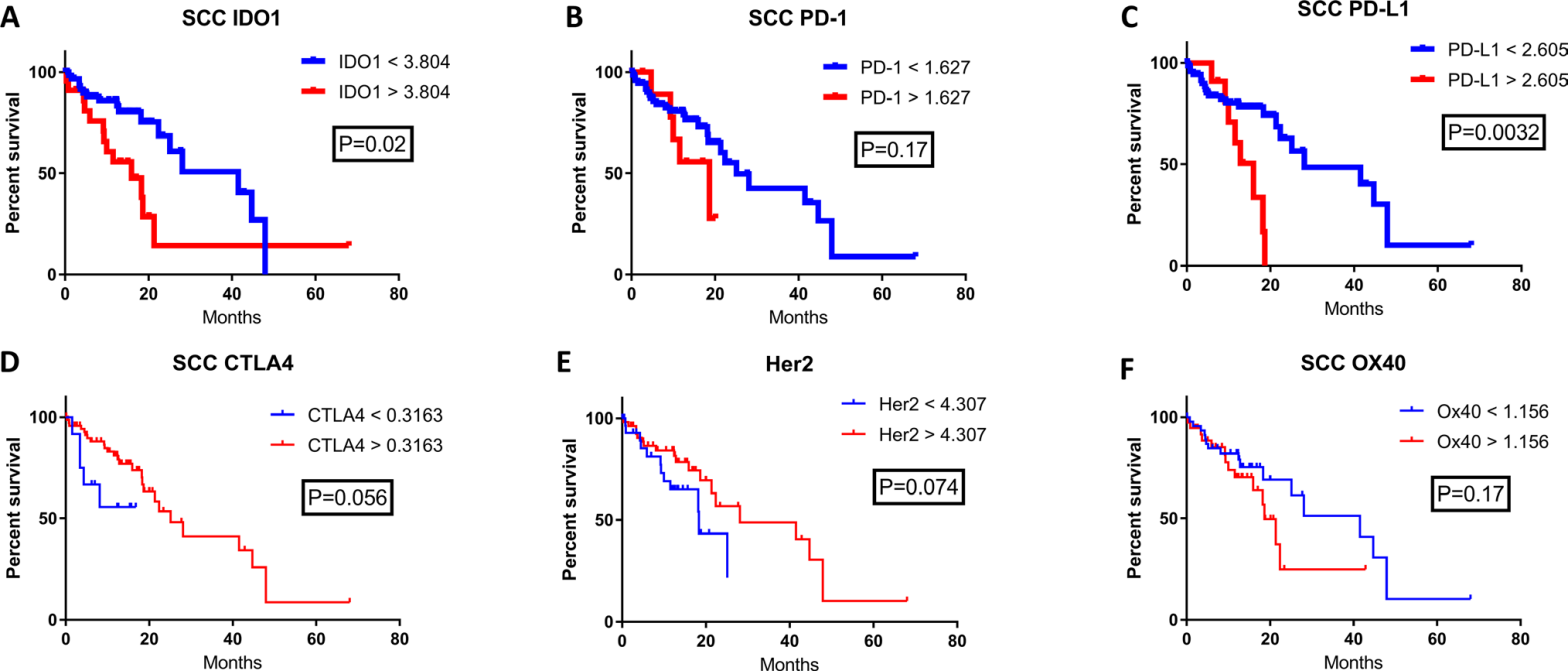
The correlation of patient survival with immune checkpoint expression mRNA levels in esophageal squamous cell carcinoma TCGA mRNA expression data was collected on 87 patients with SCC, and a Cutoff Finder software was used to define high and low levels of each marker. High (red) and low (blue) expression was correlated with OS using the Kaplan-Meier survival analysis of (**A**) IDO1; high *n* = 24, low *n* = 63, (**B**) PD-1; high *n* = 12, low *n* = 75, (**C**) PD-L1; high *n* = 12, low *n* = 75, (**D**) CTLA4; high *n* = 75, low *n* = 12, (**E**) Her2; high *n* = 56, low *n* = 31, and (**F**) OX40; high *n* = 40, low *n* = 47.

**Figure 2:**
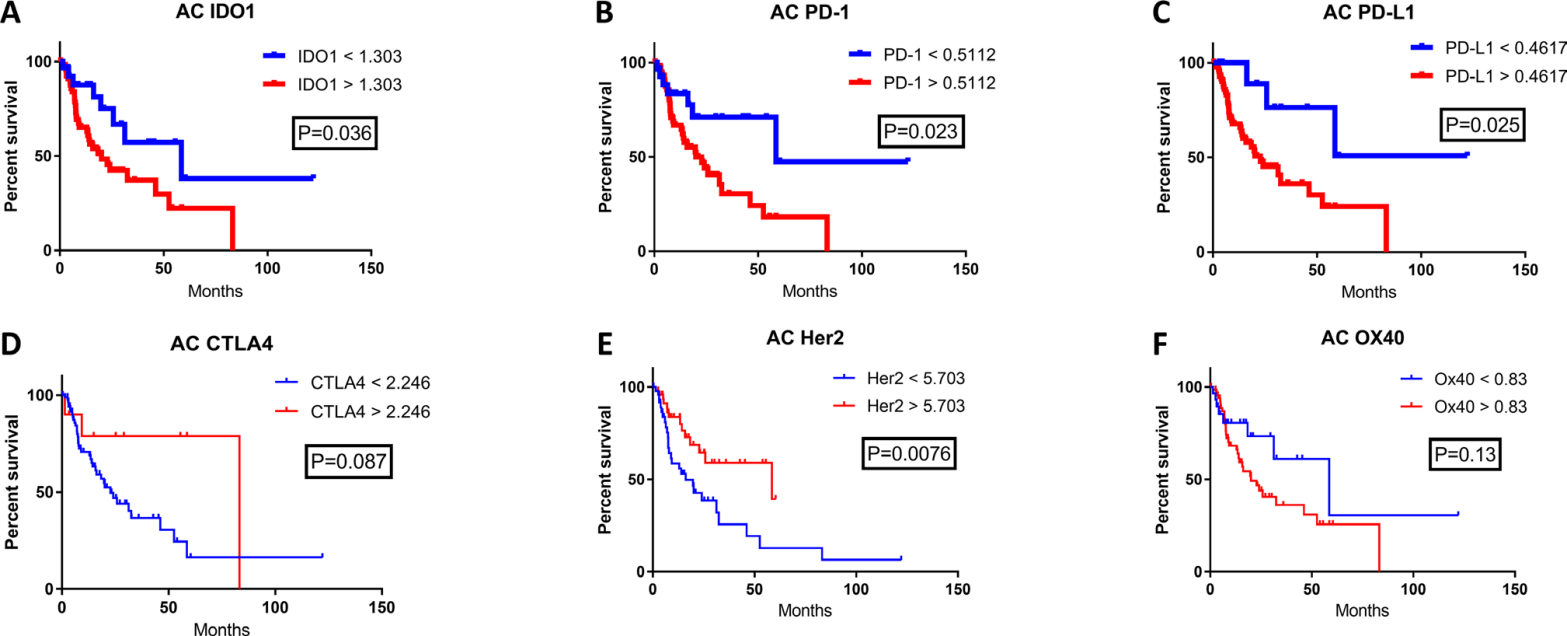
The correlation of patient survival with immune checkpoint expression mRNA levels in esophageal adenocarcinoma TCGA mRNA expression data was collected on 97 patients with esophageal adenocarcinoma, and a Cutoff Finder software was used to define high and low levels of each marker. High (red) and low (blue) expression was correlated with OS using the Kaplan-Meier survival analysis of (**A**) IDO1; high *n* = 68, low *n* = 29, (**B**) PD-1; high *n* = 69, low *n* = 28, (**C**) PD-L1; high *n* = 86, low *n* = 11, (**D**) CTLA4; high *n* = 10, low *n* = 87, (**E**) Her2; high *n* = 49, low *n* = 48, and (**F**) OX40; high *n* = 68, low *n* = 29.

Checkpoint marker expression in esophageal cancer was explored and expression was correlated with patient survival. Using mRNA expression data from TCGA for esophageal SCC and AC, expression of IDO1, PD-1, PD-L1, CTLA-4, Her2, and OX-40 was evaluated (Figures 1A–1F; 2A–2F). Expression levels by mRNA were quantified and correlated with OS. Patient samples were separated into SCC and AC based on histology for survival analysis. Cutoff Finder was utilized to generate individual cutoff values of each marker to define high and low mRNA expression. Patient survival was estimated using Kaplan-Meier analysis. The SCC cohort evaluated included a sample size of 87 patients, while the AC cohort assessed 97 patient samples.

High IDO1 mRNA levels were significantly correlated with decreased patient survival for both esophageal SCC and AC. In the SCC cohort, median OS for high versus low IDO1 mRNA levels were 15.9 months and 41.5 months respectively (Figure 1A; *P* = 0.02). Similarly, in the esophageal AC cohort, median OS was 20.1 months and 58.6 months in high and low IDO1 mRNA levels respectively (Figure 2A; *P* = 0.036).

The prognostic significance of other immune checkpoints in these patient samples were also assessed. There was a significantly shorter patient survival in patients with high PD-L1 mRNA levels when compared with low PD-L1 expression in both the esophageal SCC and AC cohorts. In the SCC cohort, the overall survival for high versus low PD-L1 expression was 15.9 months compared with 28.1 months, respectively (Figure 1C; *P* = 0.0032). In the esophageal AC patient cohort, median OS was 22.8 months for high PD-L1 mRNA levels while median OS was not reached (NR) for low PD-L1 (Figure 2C; *P* = 0.025). Interestingly, there was a statistically significant difference in OS when stratified by PD-1 expression for esophageal AC, with median OS for high and low PD-1 mRNA levels of 20.1 months and 58.6 months, respectively (Figure 2B; *P* = 0.023). Other relevant markers in esophageal cancer samples including CTLA-4, Her2, and OX40 were also evaluated for correlation with survival (Figures 1D–2F; Figures 2D–2F), however, differences in patient survival did not reach statistical significance with the exception of Her2 in AC (Figure 2E; *P* = 0.0076)

### IDO1 and PD-(L)1 co-expression is associated with decreased survival in esophageal cancer

**Figure 3:**
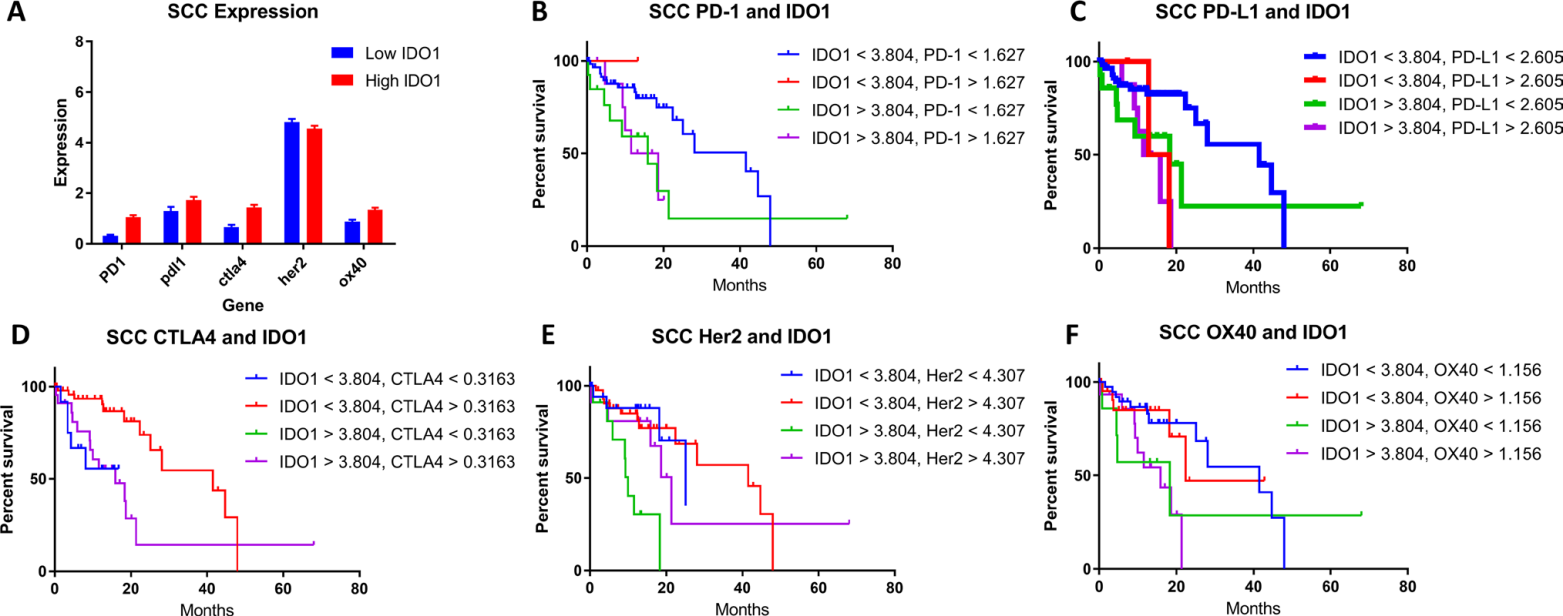
(**A**) High (red) and low (blue) IDO1 expression in patient samples stratified by gene co-expressors in SCC. The correlation of patient survival with variable IDO1 co-expression in SCC with (**B**) PD-1, (**C**) PD-L1, (**D**) CTLA-4, (**E**) Her2, and (**F**) OX-40. TCGA mRNA expression data was collected on 87 patients with esophageal SCC, and a Cutoff Finder software was used to define high and low levels of each marker in addition to IDO1. Co-expression groups for each marker were stratified in four groups: (B) High PD-1 and high IDO1, high PD-1 and low IDO1, low PD-1 and high IDO1, and low PD-1 and low IDO1. (C) High PD-L1 and high IDO1, high PD-L1 and low IDO1, low PD-L1 and high IDO1, and low PD-L1 and low IDO1. (D) High CTLA4 and high IDO1, high CTLA4 and low IDO1, low CTLA4 and high IDO1, and low CTLA4 and low IDO1. (E) High Her2 and high IDO1, high Her2 and low IDO1, low Her2 and high IDO1, and low Her2 and low IDO1. (F) High OX40 and high IDO1, high OX40 and low IDO1, low OX40 and high IDO1, and low OX40 and low IDO1. Kaplan-meier survival curves were plotted for each cohort respectively.

**Figure 4:**
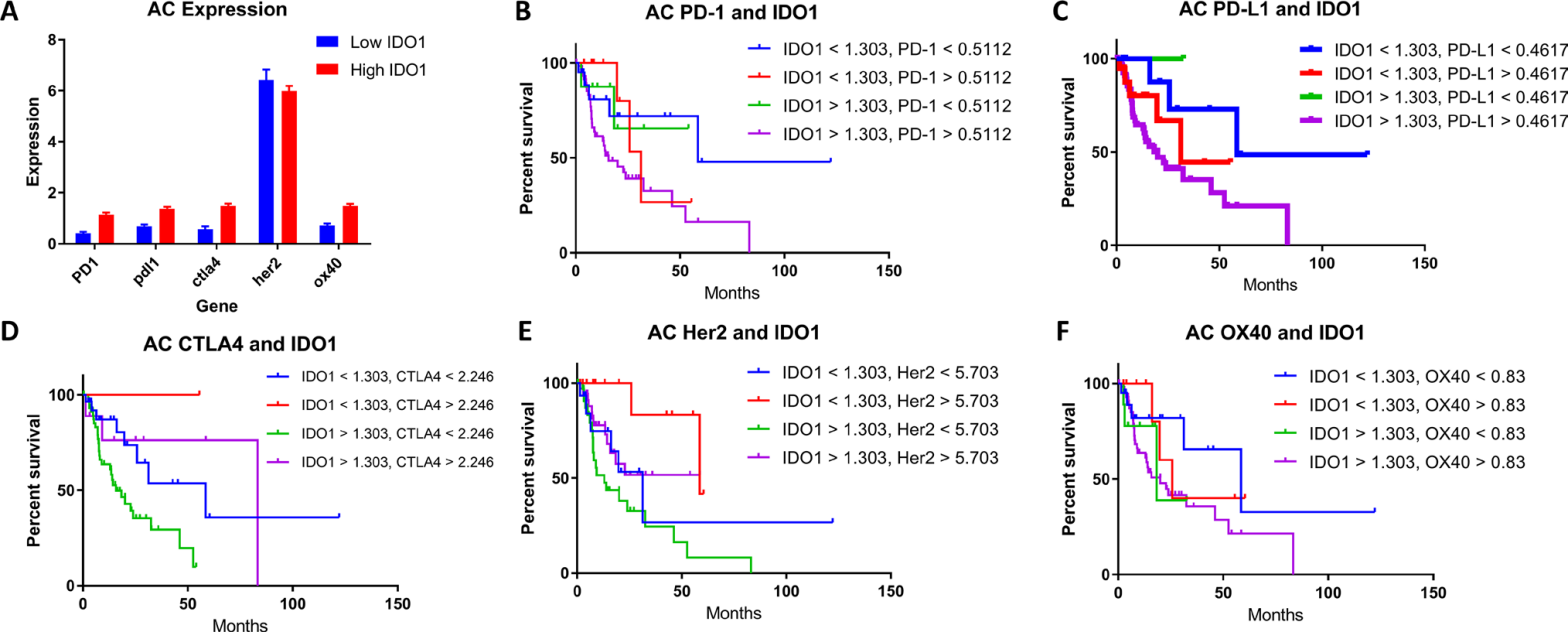
(**A**) High (red) and low (blue) IDO1 expression in patient samples stratified by genes in AC. The correlation of patient survival with variable IDO1 co-expression in AC with (**B**) PD-1, (**C**) PD-L1, (**D**) CTLA-4, (**E**) Her2, and (**F**) OX-40. TCGA mRNA expression data was collected on 97 patients with esophageal adenocarcinoma, and a Cutoff Finder software was used to define high and low levels of each marker in addition to IDO1. Co-expression groups for each marker were stratified in four groups: (B) High PD-1 and high IDO1, high PD-1 and low IDO1, low PD-1 and high IDO1, and low PD-1 and low IDO1. (C) High PD-L1 and high IDO1, high PD-L1 and low IDO1, low PD-L1 and high IDO1, and low PD-L1 and low IDO1. (D) High CTLA4 and high IDO1, high CTLA4 and low IDO1, low CTLA4 and high IDO1, and low CTLA4 and low IDO1. (E) High Her2 and high IDO1, high Her2 and low IDO1, low Her2 and high IDO1, and low Her2 and low IDO1. (F) High OX40 and high IDO1, high OX40 and low IDO1, low OX40 and high IDO1, and low OX40 and low IDO1. Kaplan-meier survival curves were plotted for each cohort respectively.

Checkpoint marker co-expression of IDO1 with PD-1, PD-L1, CTLA-4, Her2, and OX-40 was also evaluated. (Figures 3A–3F; 4A–4F). Co-expression of mRNA for IDO1 and PD-L1 was found to be correlated with patient survival in esophageal cancer. SCC patients with high IDO1 and PD-L1 co-expression compared with low IDO1 and PD-L1 co-expression possessed a median OS of 13.7 months and 41.5 months, respectively (Figure 3C; *P* = 0.0031). A similar analysis for patients with AC samples was performed. Patient samples with high IDO1 and PD-L1 levels correlated with a median overall survival of 20.2 months compared to 58.6 months in patients with low IDO1 and PD-L1 expression (Figure 4C; *P* = 0.0186).

When comparing survival in patients with co-expression of IDO1 and PD-1, similar findings for both SCC and AC histologies were identified. In SCC, patients with high IDO1 and PD-1 expression compared with low expression for these markers, median overall survival was 15.1 months and 41.5 months, respectively. (Figure 3B; *P* = 0.0151) Similarly, in AC, patients that demonstrated high versus low IDO1 and PD-1 co-expression had a median overall survival of 15.8 months and 58.6 months, respectively (Figure 4B; *P* = 0.0148).

### IDO1 has a high rate of co-expression with other checkpoint markers in esophageal cancer

To understand the correlation of IDO1 with other immunosuppressive factors in esophageal cancer, the expression levels of other genes in available esophageal cancer patient samples were stratified into IDO1-low and IDO1-high groups (Figures 3A, 4A). This comparison suggests that overexpression of PD-1, PD-L1, and CTLA-4, may be associated more frequently with high IDO1 mRNA levels compared with low IDO1 mRNA levels.

### IDO1 expression in esophageal cancer is associated with IFNɣ and IFNβ

**Figure 5:**
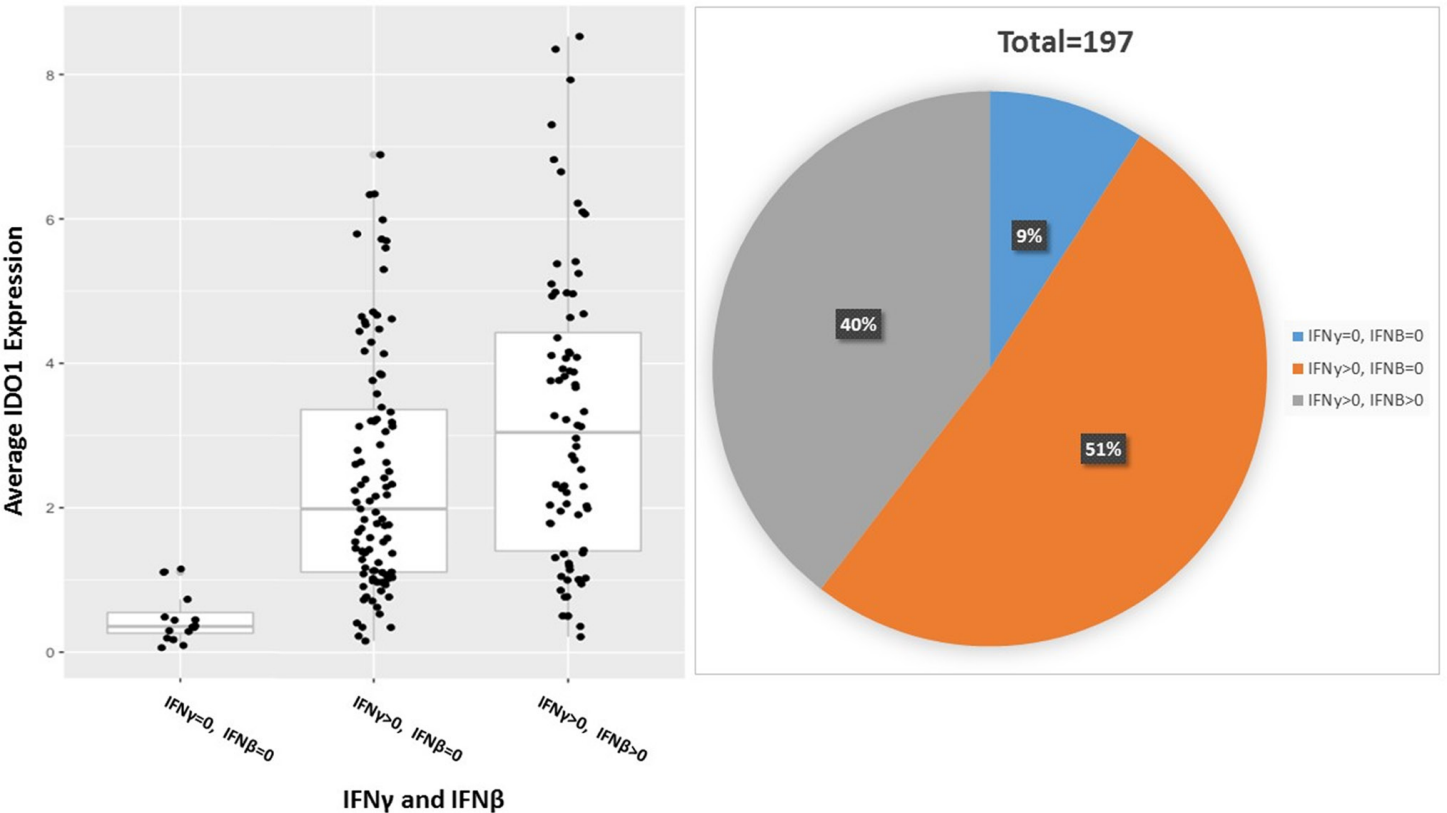
IFN-gamma and IFN-beta induce IDO1 expression in esophageal cancer; *P* = *2.2e-07* 197 total patient samples were stratified by IFNγ and IFNβ expression into 3 cohorts: IFNγ = 0 and IFNβ = 0, IFNγ > 0 and IFNβ = 0, IFNγ > 0 and IFNβ > 0. The percent of patient samples in each cohort is represented in a pie chart above.

In many tissues, IDO1 expression is undetectable [34], but rapidly induced and made detectable by pro-inflammatory stimuli [35]. Previous work has demonstrated that interferon-gamma (IFNɣ) secreted in the tumor microenvironment increases IDO1 expression, similar to PD-L1 [29, 32]. Interferon levels (IFNɣ and IFNβ) were correlated with IDO1 expression in the esophageal cancer patient samples from the TCGA (*n* = 198). Samples with undetectable IFNɣ and IFNβ (IFNɣ = 0, IFNβ = 0) was compared with detectable IFNɣ (IFNɣ > 0) and IFNβ (IFNβ > 0). 1 sample had detectable IFNβ but not IFNɣ, which was not included in analysis. Detectable IFNɣ and IFNβ correlated with increased average IDO1 expression (Figure 5; *P* = 2.2e-07).

### IDO1 protein expression is correlated with PD-L1 and CD3ε in esophageal cancer

**Figure 6:**
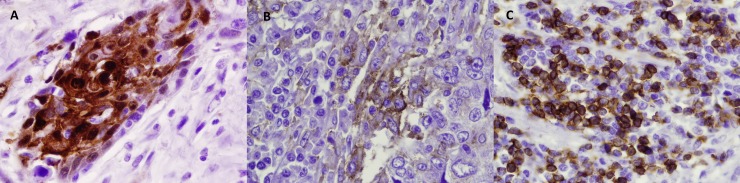
Representative IHC staining of resected esophageal SCC samples of IDO-1 (**A**), PD-L1 (**B**), and CD3ε (**C**).

**Figure 7:**
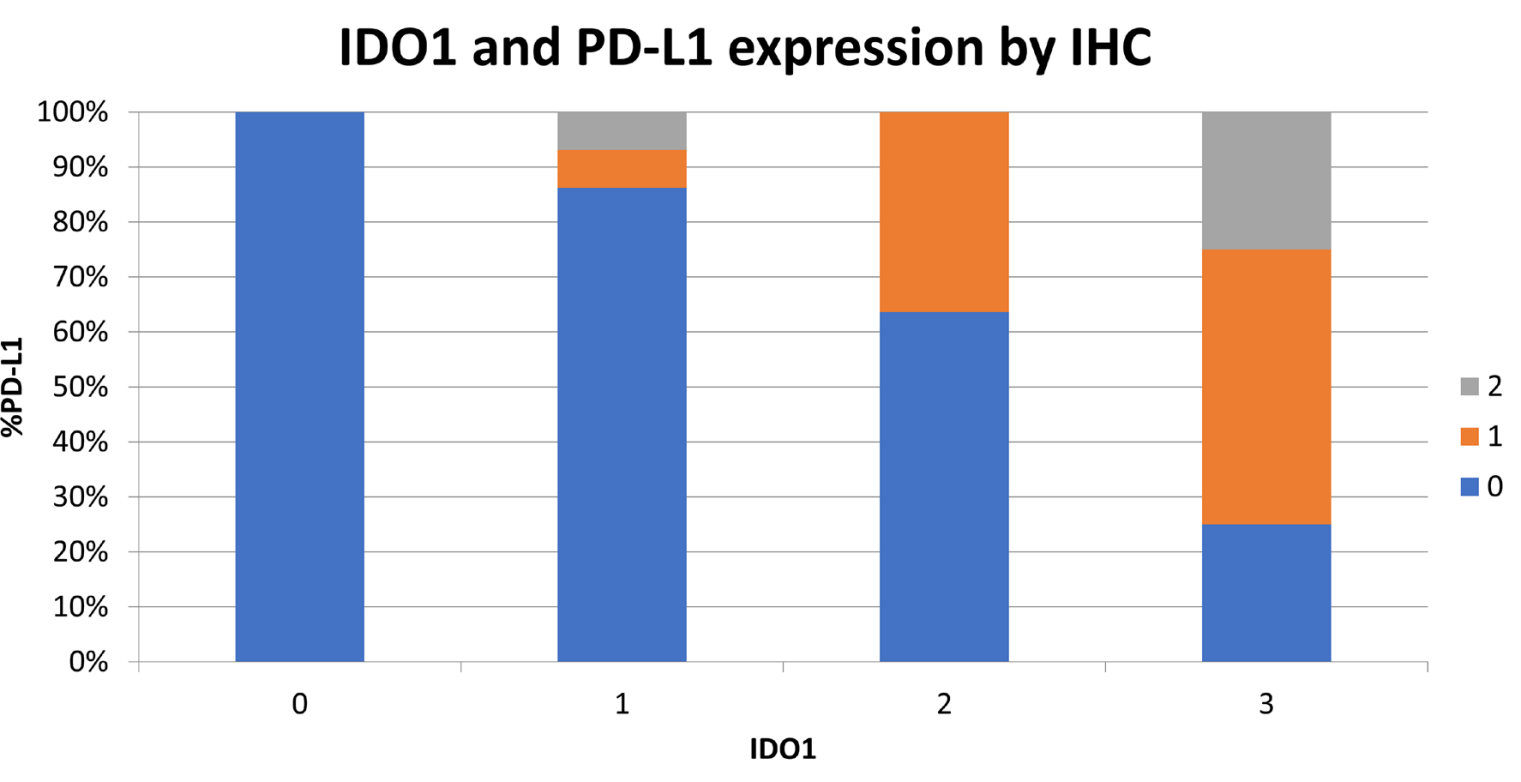
Increased IDO1 expression by IHC is correlated with increasing PD-L1 expression 93 surgically resected esophageal SCC samples were stained by IHC for IDO1 expression and PD-L1 expression on a scale of 0 to 3. Samples are shown graphically stratified by IDO1 expression demonstrating percent of PD-L1 expression on a scale of 0 to 3.

**Figure 8:**
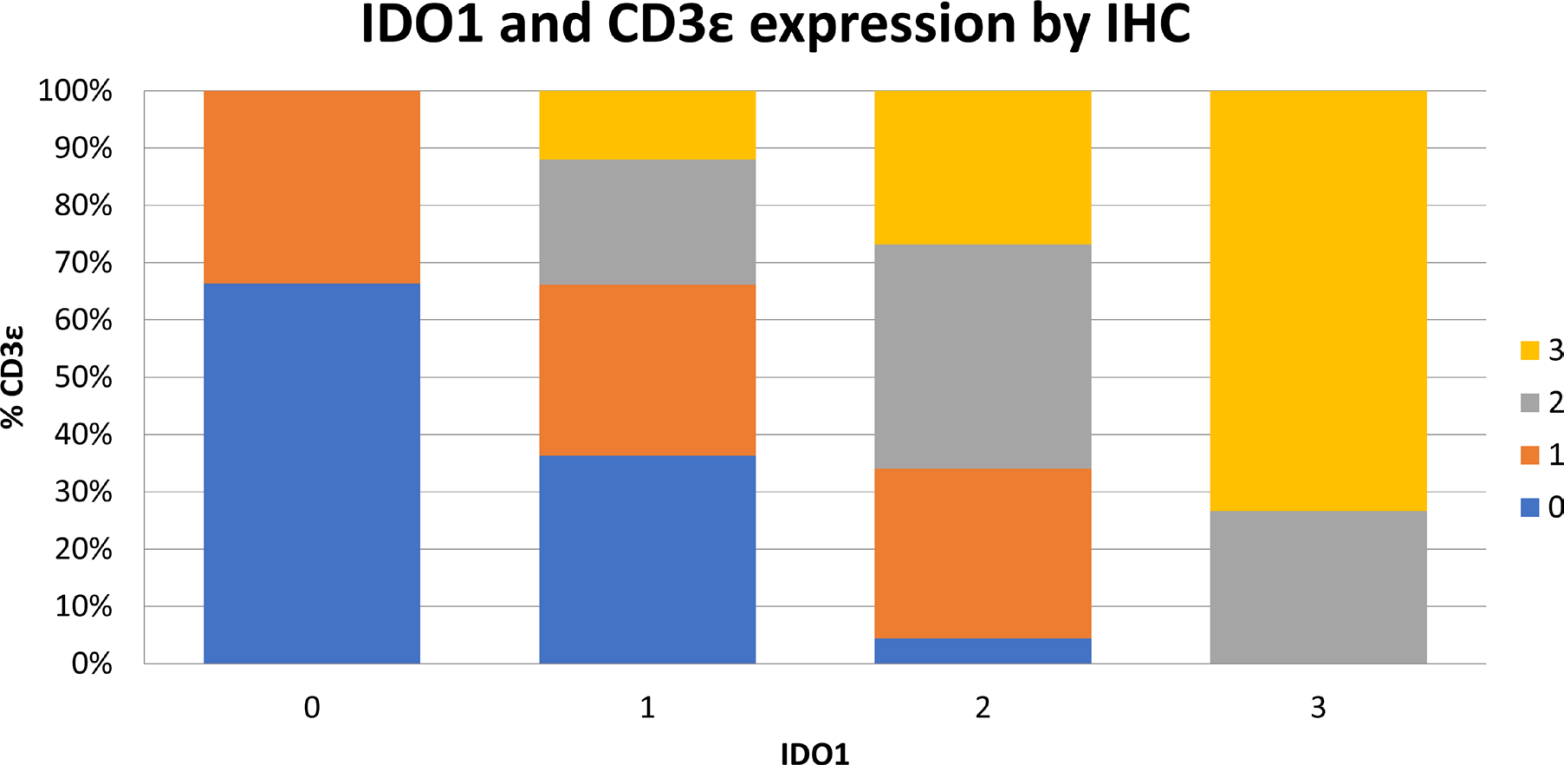
Increased IDO1 expression by IHC is correlated to increasing intratumoral CD3ε expression 93 surgically resected esophageal SCC samples were stained by IHC for IDO1 expression and CD3**ε** expression on a scale of 0 to 3. Samples are shown graphically stratified by IDO1 expression demonstrating percent of CD3ε expression on a scale of 0 to 3.

**Figure 9:**
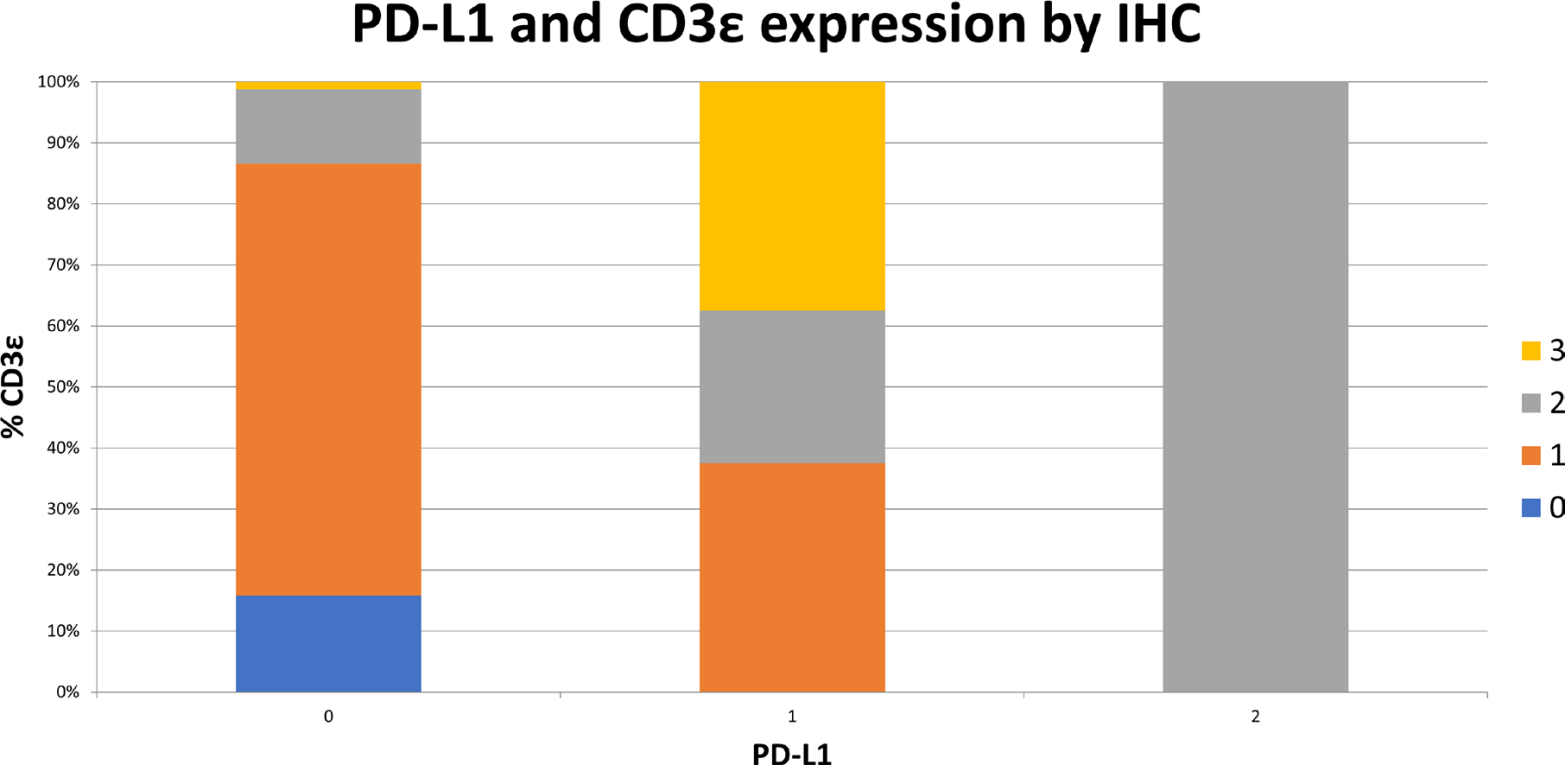
Increased PD-L1 expression by IHC is correlated to increasing intratumoral CD3ε expression 93 surgically resected esophageal SCC samples were stained by IHC for PD-L1 expression and CD3**ε** expression on a scale of 0 to 3. Samples are shown graphically stratified by PD-L1 expression demonstrating percent of CD3ε expression on a scale of 0 to 3.

To determine whether the mRNA expression data correlated with protein levels, expression by immunohistochemistry (IHC) for IDO1 was investigated among 93 surgically-resected esophageal SCC tumors evaluated in a tumor microarray (TMA). Tissue samples were stained for IDO1, PD-L1, and CD3ε, followed by light microscopic immunoscoring analysis (Figure 6) on a scale of 0 to 3. 44 of 93 samples (47.3%) stained positively for IDO1 expression. IDO1 protein expression strongly correlated with both PD-L1 (Figure 7; *P* = 0.0001), and CD3ε protein localization (Figure 8; *P* < 0.0001). The expression of PD-L1 also strongly correlated with CD3ε (Figure 9; *P* ≤ 0.0001).

IDO1 and PD-L1 expression by IHC in the surgically resected cohort was assessed for correlation with patient survival. In contrast to analysis of the TCGA data which correlated high IDO1 and PD-L1 levels with worse patient outcomes, in our surgically resected cohort expression of IDO1, PD-L1, and CD3ε by IHC did not correlate with OS.

## DISCUSSION

This investigation began with an evaluation of the prognostic role of IDO1 as a predictor of patient survival in esophageal cancer. Using mRNA gene expression of TCGA data, it was shown that increased IDO1 mRNA expression is associated with worse patient outcomes in esophageal cancer, both in SCC and AC histologies. It was further demonstrated that high PD-L1 mRNA expression, alone and in combination with high IDO1 expression, is associated with a worse overall patient survival.

While increased IDO1 expression has been associated with worse patient outcomes in a number of human malignancies [36] including esophageal SCC [37, 38], other tumors such as renal cell carcinoma, hepatocellular carcinoma, and melanoma demonstrate a correlation between IDO1 expression and improved survival [39]. This variable suggests a differential immunosuppressive role of IDO1 across tumor types. Our data support a potential negative prognostic impact of increased IDO1 expression in both SCC and AC histologies. Furthermore, these data corroborate previous reports of worsened patient outcomes in esophageal cancer with over-expression of PD-L1 and PD-L2 [40]. Previous work has suggested an association between cancer, inflammation, and increased IDO1 expression within tumors [31, 41], suggesting that similar to PD-L1, intratumoral inflammation may lead to adaptive resistance as a mechanism of tumor immune evasion within the tumor microenvironment.

This analysis also identified a correlation between IDO1 expression and the presence of IFNβ and IFNγ. This, too, is consistent with previous reports that IDO1 expression increases in response to IFNγ produced by CD8^+^ T cells within the tumor microenvironment in melanoma [32]. Taken together, these findings support the hypothesis that PD-(L)1 inhibition may lead to increased IFNγ levels as mediated by CD8^+^ T-cells within esophageal cancers. This in turn may increase IDO1 expression as an adaptive immunosuppressive mechanism, contributing to resistance of checkpoint blockade.

To further explore this hypothesis, an investigation of IDO1, PD-L1, and CD3ε (T-cell marker) expression by IHC in an additional cohort of patients with surgically resected esophageal SCC was performed. Using correlative analysis, we found a strong association between IDO1, PD-L1, and CD3ε expression. This finding suggests that increased T-cell infiltration in the tumor microenvironment is associated with high expression of IDO1 and PD-L1 in esophageal cancer. This may occur through production of pro-inflammatory cytokines such as IFNγ from T-cells within the tumor microenvironment. We hypothesize that the elevated IDO1 and PD-L1 expression in T-cell inflamed esophageal cancer lead to an immunosuppressive tumor microenvironment and contribute to worse OS. These findings suggest that IDO1 inhibition may be effective in a combinatorial approach with checkpoint inhibitor therapies in esophageal cancer. Of interest, checkpoint inhibition was recently combined with IDO1 inhibition in advanced melanoma which apparently improved response rates over single-agent PD-1 blockade [42]. This study's data suggests that similar to advanced melanoma, this may be an effective immunotherapeutic approach in esophageal cancer as well.

One limitation to this analysis is that CD3ε does not allow us to differentiate between CD8^+^ cytotoxic T cells (Tc), CD4^+^ T-helper cells (Th), and T regulatory cells (Treg). Previous work suggests that tumor cell IDO1 facilitates Treg accumulation, and that Tc and Treg infiltrate IDO1 expressing tumors, which is associated with worse survival in animal models [43–45] Additional work evaluating IDO1 in esophageal cancer could include additional staining of tumor associated T-lymphocytes for CD8 (Tc) and FoxP3 (Treg) to further elucidate the T-cell composition within IDO1 over-expressing esophageal cancer. Also, the association of worse overall survival with IDO1 expression as determined by TCGA analysis was discordant with IDO1 expression by IHC in a second cohort, which did not find an association with overall survival. Variability in these methods has been previously reported [45]. This discrepancy could be further explored with an evaluation of *in situ* hybridization or NanoString for mRNA expression, along with IHC on the same tissue samples to demonstrate discordance between methods.

In conclusion, we hypothesize based on this correlative data that in esophageal cancer, use of combinatorial immune checkpoint blockade targeting IDO1 in combination with PD-(L)1 and CTLA-4 blockade may enhance the reactivation of tumor-infiltrating T-cells, decrease immunosuppressive Tregs, and therefore amplify T-cell mediated anti-tumor responses which may further improve patient outcomes with advanced esophageal cancer.

## MATERIALS AND METHODS

### The cancer genome atlas (TCGA) sample description

The TCGA data for all the cancer types analyzed in current study were accessed from the UCSC Xena browser (http://xena.ucsc.edu/). mRNA expression data represented by RNASeq (Illumina Hi-seq platform) includes RSEM normalized level 3 data present in TCGA as of January 2017. DNA methylation data and exon expression RNASeq data were extracted from the same TCGA dataset. TCGA esophageal gene expression data by AffyU133a array were also acquired from the UCSC Xena browser.

### Immunohistochemistry (IHC) staining

Esophageal squamous cell carcinoma human paraffin embedded tissue microarrays (US Biomax, Inc.) which were labeled “HEso-Squ172Sur-02.” This consisted of 93 cases, 79 cases had tumor and matched normal adjacent tissue. Additional information available included clinical stage (I-III; AJCC 7th Ed.), survival information, surgery date (ranging from January 2009 through January 2010), with 3-4 year follow-up. The slides were incubated in decloaking chamber (Biocare Medical) at 110° C for 5 minutes; rinsed in distilled water 2 times and in 1× phosphate buffered saline (PBS) for 5 minutes, then incubated with anti-IDO1 antibody (clone: Cell Signaling antibody, #86630, clone D5J4E), anti-PD-L1 antibody (clone: Abcam, ab205921, clone 28-8) (1:50 dilution), and anti-CD3ε (clone: Abcam, ab16669, clone SP7) (1:50 dilution) in antibody diluent (DAKO, Cat# S0809) overnight at 4° C. After rinsing with Tris-Buffered NaCl Solution with 0.1% Tween 20 (TBST) (DAKO), sections were further incubated with HRP-labelled anti-rabbit secondary antibody (DAKO, Cat# K4011) for 30 minutes. Slides were then washed for 3 minutes. Immunohistochemical reactions were visualized using EnVision System-HRP (DAKO). Tissue sections were counterstained with hematoxylin Gill II (Surgipath), mounted in the mounting medium, and visualized under a light microscope. Quantitative IHC immunoscoring was subsequently performed by Victoria Villaflor, MD at Northwestern University.

### Statistical analysis

The cutoff value for each gene expression level was determined with Cutoff Finder software (http://molpath.charite.de/cutoff/) using significance as the cutoff optimization method [25]. Kaplan-Meier (KM) survival analysis was performed to estimate the survival distribution, while the log-rank test was used to assess the statistical significance of differences between the stratified survival groups using GraphPad Prism (version 6, GraphPad Software, Inc., La Jolla, CA). To assess statistical differences between stratified survival groups for co-expression curves, the log-rank test for trend was used. To assess correlation of IFNγ and IFNβ with IDO1 levels, Kendall's rank correlation tau was used. Differences were considered to be statistically significant when *P* < 0.05. IHC staining of IDO1, PD-L1, and CD3ε of esophageal SCC samples were correlated by Fisher's exact test. Differences were considered statistically significant when *P* < 0.05.

## Abbreviations

IDO1: Indoleamine 2,3-dioxygenase 1
TCGA: the cancer genome atlas
IHC: immunohistochemistry
SCC: squamous cell carcinoma
AC: adenocarcinoma
PD-L1: programmed death ligand-1
GEJ: gastroesophageal junction
CTLA-4: cytotoxic T lymphocyte antigen-4
MSI-high: microsatellite instability – high
PFS: progression free survival
OS: overall survival
GCN2: general control non-depressible 2
